# Diagnostic performance of real-time characterization in artificial intelligence-assisted colonoscopy

**DOI:** 10.1055/a-2860-7702

**Published:** 2026-06-05

**Authors:** Ronja M. B. Lagström, Karoline B. Bräuner, Mikkel N. Frandsen, Ismail Gögenur, Mustafa Bulut

**Affiliations:** 1 Department of Surgery 524788 Zealand University Hospital KogeKøgeSjællandDenmark; 2 Department of Surgery 73014 Slagelse HospitalSlagelseRegion SjællandDenmark; 3 Faculty of Health and Medical SciencesDepartment of Clinical Medicine 53139 University of CopenhagenCopenhagenDenmark; 4 Copenhagen Academy for Medical Education and Simulation (CAMES)CopenhagenDenmark

**Keywords:** Endoscopy Lower GI Tract, Polyps / adenomas / ..., CRC screening, Diagnosis and imaging (inc chromoendoscopy, NBI, iSCAN, FICE, CLE...)

## Abstract

**Background and study aims:**

Because most colorectal polyps are diminutive and carry minimal cancer risk, artificial intelligence (AI) might enable diagnostic strategies such as leave-in-situ and resect-and-discard, provided it meets predefined quality benchmarks. We assessed diagnostic performance of a computer-aided polyp characterization system (CADx) in predicting histology of diminutive rectosigmoid polyps during colonoscopy in a Danish population.

**Patients and methods:**

We conducted a prospective diagnostic accuracy study across four endoscopy centers. Adults referred for colonoscopy due to a positive fecal immunochemical test (FIT), surveillance, or other diagnostic indications were included. Patients with inadequate bowel preparation were excluded. Histopathology served as the reference standard and all polyps were categorized as adenomas or non-adenomas. Several performance metrics are reported.

**Results:**

We included 278 patients and a total of 772 polypectomies for analysis. For diminutive rectosigmoid polyps (n = 184), the CADx-system achieved a sensitivity of 93% (95% confidence interval [CI] 87%-97%) and a specificity of 33% (95% CI 22%-46%). Positive and negative predictive values were 70% (95% CI 62%-77%) and 74% (95% CI 55%-88%), respectively, with an overall accuracy of 71% (95% CI 64%-77%) and a diagnostic odds ratio of 6.69.

**Conclusions:**

The CADx-system demonstrated high sensitivity, but significantly lower specificity compared with prior studies, driven by a high false-positive rate. Inconsistent findings across studies highlight the challenges in standardizing AI-based characterization systems. Our results indicates that CADx is a promising future adjunct to colonoscopy, but its current performance is insufficient for reliable implementation in resect-and-discard or leave-in-situ strategies.

## Introduction

Artificial intelligence (AI) assistance during colonoscopy increases the polyp detection rate and the adenoma detection rate (ADR) 1 . The current standard of care is to remove all polyps for histopathological examination, except in the case of multiple diminutive hyperplastic polyps in the rectosigmoid colon. This, however, leads to significant costs associated with pathological assessment of polyps of limited clinical relevance 2 . Because of this uncritical removal of all polyps, more than 2,000 diminutive polyps must be removed to prevent a single case of colorectal cancer (CRC) 3 .

The European Society of Gastrointestinal Endoscopy (ESGE) has defined competence standards for optical diagnosis of diminutive polyps (1–5 mm) in the rectosigmoid colon, where assessment by a pathologist is not necessary. They suggest that implementing a leave-in-situ strategy for diminutive polyps is clinically acceptable if optical diagnosis achieves a sensitivity of 90% and a specificity of 80%. To implement a resect-and-discard strategy, both the sensitivity and specificity should be at least 80% 4 . The American Society for Gastrointestinal Endoscopy (ASGE) recommends a negative predictive value (NPV) of at least 90% for a device to guide leaving diminutive hyperplastic rectosigmoid polyps in situ 2 .

Most colorectal polyps are small and unlikely to develop into cancer, supporting safety of the resect-and-discard strategy 5 . Computer-aided polyp diagnosis (CADx) during colonoscopy holds great potential. It can increase the confidence of the endoscopist, helping with optical diagnosis of colorectal polyps 6 . Due to the varying results in the existing literature, assessment of performance of CADx in diverse populations is essential to ensure its diagnostic value and generalizability 7 8 . The aim of this study was to investigate diagnostic performance of a CADx system in predicting polyp histology during real-time colonoscopy in a Danish population.

## Patients and methods

This was a substudy of a quasi-randomized controlled trial 9 , designed as a diagnostic accuracy study to evaluate performance of the CADx system using prospectively collected data. Randomization occurred at the center level across four centers, with 2-week alternating cycles between AI-assisted and standard colonoscopy until the required sample size was reached. For the current analysis, we focused solely on data from the CADx arm (n = 399).

### Eligibility

All patients included were aged 18 years or older. They were referred either for CRC screening following a positive fecal immunochemical test (FIT) (> 100 μg/L), surveillance, or diagnostic colonoscopy at four centers in Region Zealand, Denmark. Patients referred for removal of previously detected polyps, patients referred for control colonoscopies due to inflammatory bowel disease, and emergency colonoscopies were not included in the trial. Bowel preparation was evaluated using a modified version of the Aronchick scale; graded as good, suboptimal, or poor – indication for new colonoscopy 10 . Only colonoscopies with good and suboptimal bowel preparation were included. Procedures were only included in the study if the cecum/ileocolonic anastomosis was intubated. We only included colonoscopies where at least one polyp was found and removed.

### Artificial intelligence system

We used the AI system GI Genius (generation 3, Medtronic), a combined computer-aided detection (CADe) and diagnosis system (CADx). This system serves as a supplement to conventional video colonoscopy, designed to analyze colonoscopy images in real time. The system operates independently of the colonoscopy monitor and evaluates each polyp as an isolated input.

**Fig. 1:**
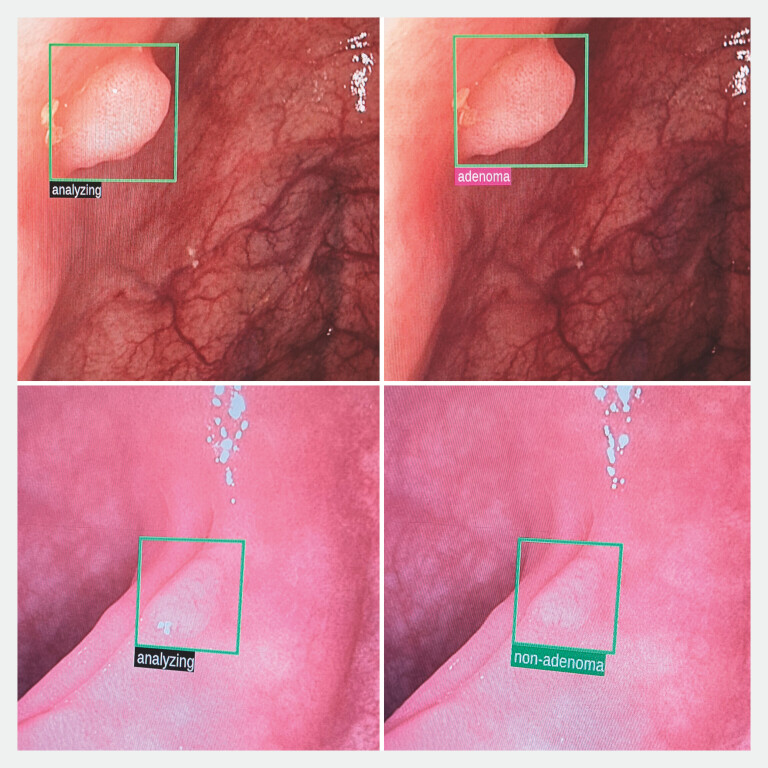
Two polyps are detected by the combined CADe- and CADx-system. The first polyp is characterized as an adenoma, and the second one is characterized as a non-adenoma

It marks lesions with visual features that may correspond to various mucosal abnormalities. Detected lesions are instantly highlighted on the screen and classified as adenoma, non-adenoma, or no prediction ( Fig. 1 ). All procedures were performed with a high-definition EXERA III setup (Olympus CF-HQ190L and CLV/CV-190; Olympus Co., Tokyo, Japan) and only white light was used for purpose for the study.

### Procedures and polyps

The procedures were carried out by endoscopists with different levels of expertise. Colonoscopies were carried out by both non-expert (under 1000 colonoscopies) and expert (more than 1000) endoscopists. Macroscopic characteristics of all polyps detected during the procedure were reported in patient charts, as well as real-time characterization made by CADx. Endoscopist prediction of histopathology was not documented.

All polyps were histopathologically divided into two groups: adenomas and non-adenomas. Adenomas were defined as tubular and tubulovillous adenomas with low- and high-grade neoplasia. Sessile serrated lesions (SSLs) with dysplasia and cancerous polyps were also considered adenomas. Non-adenomas were defined as hyperplastic polyps, SSLs without dysplasia, normal tissue, and other polyps without dysplasia.

Our primary outcomes were sensitivity and specificity of the CADx system alone, without human interaction, calculated from the prespecified counts of true positives (TPs), true negatives (TNs), false positives (FPs), and false negatives (FNs) based on comparison with the reference standard, as defined in the trial registry. We also report several other classification performance metrics.

Histopathological findings were considered the gold standard for validating diagnostic accuracy of the AI system.

### Statistical analysis

Because this was a substudy, we did not perform a formal sample size calculation. To evaluate performance of the CADx system in classifying polyps, a confusion matrix was constructed. The confusion matrix categorizes classification outcomes into four groups: TP, TN, FP, and FN.

Adenomas that were missing, not identified by the system, or classified as no prediction were not included in the primary analysis.

From the confusion matrix, the following metrics were calculated to assess AI diagnostic performance: 1) sensitivity (TP rate), defined as the proportion of actual positives that were correctly identified by the AI system; 2) specificity (TN rate), defined as the proportion of actual negatives that were correctly identified; 3) positive predictive value (PPV), defined as the proportion of positive identifications that were correct; 4) negative predictive value, defined as the proportion of negative identifications that were correct; 5) accuracy, defined as the overall proportion of correct predictions; 6) no information rate (NIR), defined as the proportion of correct prediction with naïve classification, meaning predicting the majority group in all cases, calculated as $\frac{\left(TP+FN\right)}{\left(TP\;+TN+FP+FN\right)}$ . This is not a performance metric of the classification system but gives information in relation to the accuracy; and 7) diagnostic odds ratio (DOR), a measure of overall performance of a diagnostic test, calculated as $\frac{\left(TP/FN\right)}{\left(FP/TN\right)}$ = $\frac{sensitivity/(1-sensitivity)}{(1-specificity)/specificity}$

Differences between FP and FN proportions were assessed with McNemar’s test. Agreement between CADx and histopathology was evaluated using Cohen’s κ.

We also decided to do a clinical sensitivity analysis, based on the assumption that all polyps that CADx could not assess would be removed if the resect-and-discard or leave-in-situ strategies would be implemented. In this analysis no prediction and not detected, therefore, were labelled as adenomas.

All statistical analyses were performed using R software version 4.2.1 (2022–06–23 ucrt), utilizing the dplyr package for data manipulation. All polyps were treated as independent from each other despite study participants contributing several polyps to the analyses. Confidence intervals (Cis) for the total number of polyps were calculated using the z-test for proportions, whereas for smaller groups, the binomial test (Clopper-Pearson) for proportions was applied. The significance level was arbitrarily set at 0.05.

## Results

### Procedures

We had a cohort of 399 patients, with an ADR of 59.1%. There were 67 procedures (16.8%) performed by non-expert endoscopists and 332 (83.2%) by experts, and 160 (40.1%) of the procedures were CRC screenings due to a positive FIT test. Of this cohort, we included the 278 patients from whom at least one polyp was removed.

### Polyp characteristics

There were a total of 772 polypectomies. In the rectosigmoid colon, 294 polyps were removed with 221 being diminutive (1–5 mm). One polyp was adenocarcinogenic ( Table 1 ).

**Table:** **Table 1** Polyp characteristics.

Characteristics	n
**Polyps**	772
**Localization n (%)**	
Cecum	65 (8.4)
Ascending colon	160 (20.7)
Right flexure	30 (3.9)
Transverse colon	131 (17.0)
Left flexure	28 (3.6)
Descending colon	64 (8.3)
Sigmoid colon	193 (25.0)
Rectum	101 (13.1)
**Polyp type n (%)**	
Polypoid	692 (89.6)
Non-polypoid	80 (10.4)
**Size polyps n**	
1–5 mm	613 (79.4)
1–5 mm in the rectosigmoid colon	221 (28.6)
6–9 mm	84 (10.9)
≥ 10 mm	75 (9.7)
**Size adenomas n**	
1–5 mm	413 (76.6)
6–9 mm	63 (11.7)
≥ 10 mm	63 (11.7)
**Histopathology**	
**Adenoma**	539
Tubular adenoma low-grade	517 (95.9)
Tubular adenoma high-grade	9 (1.7)
Tubulovillous adenoma low-grade	4 (0.7)
Tubulovillous adenoma high-grade	1 (0.2)
Sessile serrate lesion with dysplasia/sessile serrate adenoma	7 (1.3)
Adenocarcinoma	1 (0.2)
**Non-adenoma**	233
Hyperplasia	82 (35.2)
Sessile serrate lesion without dysplasia	89 (38.2)
Normal tissue	34 (14.6)
Granulation tissue	2 (0.9)
Inflammatory polyp	7 (3.0)
Other (benign)	19 (8.2)

## Performance of the CADx system

Distribution of outcomes by CADx among different sized polyps in colon is shown in Table 2 . A total of 83 polyps were classified as no prediction and 12 were not detected by the system ( Table 3 ). The no prediction rate did not differ significantly between experts and non-experts (10.6% [68/643] vs 14.0% [15/107], *P* = 0.293).

**Table:** **Table 2** Distribution of outcomes by CADx among different sized polyps (total number of polyps, n = 772).

	1–5 mm	6–9 mm	≥ 10 mm	1–5 mm (rectosigmoid colon)	All polyps
True positive	358	61	56	107	475
False negative	14	0	0	8	14
False positive	98	13	3	46	114
True negative	54	6	4	23	64
No prediction	78	3	2	33	83
Not detected	8	0	4	1	12
Missing	3	1	6	3	10
CADx, computer-aided diagnosis.					

**Table:** **Table 3** Characteristics of polyps not characterized or detected by CADx.

	No prediction (n = 83)	Not detected (n = 12)
**Histopathology**	**n (%)**	
Tubular adenoma low-grade	37 (44.6)	7 (58.3)
Hyperplasia	13 (15.7)	0
Sessile serrate lesion without dysplasia	25 (30.1)	3 (25.0)
Normal tissue	4 (4.8)	1 (8.3)
Inflammatory polyp	2 (2.4)	0
Other (benign)	2 (2.4)	1 (8.3)
**Localization**	**n (%)**	
Cecum	4 (4.8)	4 (33.3)
Ascending colon	13 (15.7)	4 (33.3)
Right flexure	3 (3.6)	0
Transverse colon	14 (16.9)	0
Left flexure	4 (4.8)	1 (8.3)
Descending colon	11 (13.3)	2 (16.7)
Sigmoid colon	19 (22.9)	1 (8.3)
Rectum	15 (18.1)	0
CADx, computer-aided diagnosis		

### Diminutive polyps in the rectosigmoid colon

The CADx system predicted histology of diminutive rectosigmoid polyps (n = 184) with a sensitivity of 0.93 (95% CI 0.87–0.97) and a specificity of 0.33 (95% CI 0.22–0.46). Overall accuracy was 0.71 (95% CI 0.64–0.77). This was significantly different ( *P* = 0.013) from the NIR at 0.63 (95% CI 0.55–0.69). PPVs and NPVs were 0.70 (95% CI 0.62–0.77) and 0.74 (95% CI 0.55–0.88), respectively. The kappa value measuring agreement between CADx and histopathological classification showed only “fair” agreement with a value of 0.296, indicating limited agreement beyond chance. McNemar’s test showed that distribution of FP and FN was skewed ( *P* < 0.001), with more FPs than FNs (48 vs. 8). We calculated a DOR of 6.69 (95% CI 2.79–16.05) as moderately effective.

### All diminutive polyps

We also looked at all diminutive polyps (1–5 mm) in all colon segments (n = 524). Accuracy was 0.79 (95% CI 0.75–0.82). We found a sensitivity of 0.96 (95% CI 0.94–0.98) and a specificity of 0.36 (95% CI 0.28–0.44). The PPV and NPV were 0.79 (95% CI 0.74–0.82) and 0.79 (95% CI 0.68–0.88), respectively.

### All polyps

When including all polyps (n = 667) in our calculations, we found a sensitivity of 0.97 (95% CI 0.95–0.98) and a specificity of 0.36 (95% CI 0.29–0.44). Accuracy was 0.81 (95% CI 0.78–0.84) and NIR was 0.73 (95% CI: 0.70–0.77), with a significant difference between the two outcomes ( *P* < 0.001). PPV was 0.81 (95% CI 0.77–0.84) and NPV 0.82 (95% CI 0.71–0.89).

We reran the same analyses on all polyps in a clinical sensitivity analysis. Sensitivity was 0.94 (95% CI 0.88–0.97) and specificity was 0.24 (95% CI 0.16–0.34). PPV was 0.62 (95% CI 0.54–0.69) and NPV was 0.74 (95% CI 0.55–0.88). Accuracy was 0.63 (95% CI 0.57 - 0.68) and NIR was 0.56 (95% CI: 0.49 - 0.63), with a significant difference between the two outcomes ( *P* = 0.02).

### Characteristics of sessile serrate lesions without dysplasia

We looked at SSLs without dysplasia separately. Thirty-four of the lesions (38.2%) were correctly assessed as non-adenomas by CADx and 24 (27.0%) were assessed as adenomas. There were 25 (28.0%) without prediction, and three (3.4%) which the system failed to detect. There were missing CADx-data for three of them.

## Discussion

Computer-aided polyp characterization has potential to revolutionize polyp management during colonoscopy, provided that its diagnostic performance is sufficiently high and reliable.

A simulation model demonstrated that a resect-and-discard strategy, supported by narrow-band imaging for diagnosing diminutive polyps (1–5 mm) in screening colonoscopy, could yield substantial cost savings without affecting efficacy, primarily by reducing unnecessary pathological examinations 11 . In addition, a leave-in-situ strategy would not only reduce costs but also minimize risks associated with polypectomy 2 .

In this study, we tested the CADx system in a heterogenous group of patients with a relatively high ADR, which is relevant because ADR is a key quality indicator in colonoscopy. Most of the procedures were CRC screening colonoscopies due to a positive FIT test. The largest proportion of polyps (79%) in our study were diminutive. We calculated performance metrics given that the polyps were removed, and the CADx system had characterized them as adenomas or a non-adenomas.

Sensitivity (93%), specificity (33%), and NPV (74%) of the CADx system for optical diagnosis of diminutive polyps in the rectosigmoid colon did not meet recommended ESGE and ASGE thresholds 2 4 . Performance in characterizing diminutive polyps throughout the entire colon was slightly better but still below recommended thresholds. In our clinical sensitivity analysis, we assumed that all polyps in the colon, which CADx failed to assess, would be removed and thus technically labelled as adenomas. The NPV remained the same, but we saw even lower specificity (24%) and slightly higher sensitivity (94%). Still, the system was significantly better than just guessing the majority proportion because the accuracy was higher than the NIR.

SSLs are generally more challenging to detect than conventional adenomas. Effective colonoscopy depends on the endoscopist being adequately trained to recognize these lesions 12 . In this study, the CADx system faced challenges in characterization of SSLs without dysplasia: 38.2% were correctly identified as non-adenomas, whereas 27.0% were misclassified as adenomas, and 28.0% received no prediction. One of the features of SSLs that is associated with advanced neoplasia is proximal colon location 12 . Sessile polyps should be completely removed, except in cases with multiple diminutive lesions in the sigmoid colon or rectum that are suspected to be hyperplastic polyps 13 . However, the CADx system evaluated in this study does not incorporate lesion location and, therefore, cannot assess clinical importance of resection, which is greater for lesions in the proximal colon.

Multiple studies have assessed performance of various CADx-systems in polyp characterization, with mixed findings. In one study, the CADx system EndoBRAIN was evaluated. When used to assist endoscopists in polyp assessment, it met both ESGE and ASGE thresholds for the leave-in-situ strategy, achieving sensitivity of 90.4%, specificity of 85.9%, and NPV of 92.8% for diminutive polyps in the rectosigmoid colon. The system showed a nonsignificant increase in performance metrics for optical diagnosis compared with procedures without CADx 14 . Results from another CADx study demonstrated an improvement in specificity, but not in sensitivity 15 . A validation study of the novel system WISE VISION showed that the system met the thresholds for both resect-and-discard and leave-in-situ, independent of which endoscopy platform was used 16 .

GI Genius was tested on 295 diminutive polyps in another study, which showed NPV of 97.6%, sensitivity of 82%, and specificity of 93.2% for AI alone 7 . This indicates that the system meets the ASGE threshold for leave-in-situ and the ESGE threshold for resect-and-discard. Two other studies on GI Genius reported relatively high sensitivity and specificity; however, these values did not meet the criteria for the resect-and-discard strategy 8 17 .

Another system called CAD-EYE has shown to have a NPV of 91.1% when used for AI-assisted optical diagnosis, meeting the ASGE threshold for leave-in-situ. However, when tested without human interaction, it only showed NPV of 86.7%. Sensitivity and specificity for AI alone were 81.9% and 88.7% respectively, meeting recommendations from ESGE for resect-and discard, but not for leave-in-situ 18 . Correspondingly, CAD-EYE showed NPV of 80.6% in another clinical study. In this study, SSLs and inflammatory polyps were excluded from analysis 19 .

Specificity of CADx in our cohort is considerably lower than in other studies. Studies have shown that diminutive polyps are often misdiagnosed as normal mucosa by pathologists 20 21 . These errors can occur due to fragmentation during retrieval and processing of the polyp, potentially leading to collection and analysis of only normal colorectal epithelium or suboptimal sectioning. As a result, reliability of histopathological analysis as the gold standard for diagnosing diminutive colorectal polyps has been questioned, particularly when high-confidence optical diagnosis indicates presence of an adenoma 22 . Although variations in pathological assessment may contribute to discrepancies, they may not fully account for the substantial differences observed. Moreover, the possibility of publication bias favoring positive results cannot be ruled out.

Although specificity was low, accuracy was significantly higher than the NIR, meaning that the system performs better than just guessing the majority group every time, and the calculated DOR of 6.69 suggests that CADx has “moderate” strength in predicting correct outcomes. When calculating DORs required by ESGE, we found that DORs of 16 and 36 would be required for the resect-and-discard and leave-in-situ strategies, respectively.

A major limitation of this study is the relatively small sample size compared with previous studies, which was due, in part, to the study being a substudy of a previously sample size-calculated study. A high rate of “no prediction” outcomes was observed, with no evidence of dependence on endoscopist level of experience.

We only tested the AI diagnosis alone, and not the AI-assisted optical diagnosis, where the endoscopist is using CADx to determine the most likely pathology of the polyp. This distinction is important when comparing outcomes from different studies. It has been shown that hybrid intelligence, where humans interact with AI, is superior to AI alone when optical diagnosis of diminutive polyps is made 23 . CADx has the potential to be particularly beneficial for non-experts, enhancing their sensitivity to a level comparable to that of experts 24 . However, the value of CADx in clinical practice is still questionable because CADx has not shown any benefit in the resect-and-discard strategy 25 .

One limitation of this study is use of pathologist diagnosis as the reference standard in all cases, rather than having expert endoscopists or pathologists review images of lesions diagnosed as normal tissue —an approach applied in a previous study 20 . One of the strengths of this study is that we report a more complete picture of model performance compared with other studies, using several additional meaningful metrics. We suggest that future studies evaluating AI -enhanced clinical classification systems consider not only accuracy but also NIR and DOR, given that accuracy is influenced by class distribution.

It has been shown that CADx influences endoscopist diagnostic accuracy by enhancing it when providing the correct diagnosis and impairing it when presenting the incorrect diagnosis 26 . Our results indicate that CADx is a promising tool; however, diagnostic performance of the currently used system is insufficient for routine use in resect-and-discard and leave-in-situ strategies. However, the technology is advancing rapidly, with the latest systems often being trained on substantially larger image datasets.

## Conclusions

Our findings contribute to the growing body of knowledge on computer-aided adenoma characterization. Due to the lack of consensus in the literature regarding performance, further studies are still needed. AI-assisted colonoscopy is expected to advance rapidly, necessitating further clinical trials to evaluate emerging technologies. Future studies should also carefully consider how the diagnostic gold standard is established because misdiagnosis by pathologists may influence evaluation of diagnostic performance of an AI system.

## References

[1] Ashat M Klair JS Singh D Impact of real-time use of artificial intelligence in improving adenoma detection during colonoscopy: A systematic review and meta-analysis Endosc Int Open 2021 9 E513 E521 33816771 10.1055/a-1341-0457 PMC7969136

[2] Rex DK Kahi C O’Brien M The American Society for Gastrointestinal Endoscopy PIVI (Preservation and Incorporation of Valuable Endoscopic Innovations) on real-time endoscopic assessment of the histology of diminutive colorectal polyps Gastrointest Endosc 2011 73 419 422 10.1016/j.gie.2011.01.023 21353837

[3] Pickhardt PJ Hassan C Laghi A Small and diminutive polyps detected at screening CT colonography: a decision analysis for referral to colonoscopy AJR Am J Roentgenol 2008 190 136 144 10.2214/AJR.07.2646 18094303

[4] Houwen BBSL Hassan C Coupé VMH Definition of competence standards for optical diagnosis of diminutive colorectal polyps: European Society of Gastrointestinal Endoscopy (ESGE) Position Statement Endoscopy 2022 54 88 99 34872120 10.1055/a-1689-5130

[5] Ponugoti PL Cummings OW Rex DK Risk of cancer in small and diminutive colorectal polyps Dig Liver Dis 2017 49 34 37 10.1016/j.dld.2016.06.025 27443490

[6] El Zoghbi M Shaukat A Hassan C Artificial intelligence-assisted optical diagnosis: a comprehensive review of its role in leave-in-situ and resect-and-discard strategies in colonoscopy Clin Transl Gastroenterol 2023 14 e00640 10.14309/ctg.0000000000000640 PMC10584286 37747097

[7] Hassan C Balsamo G Lorenzetti R Artificial intelligence allows leaving-in-situ colorectal polyps Clin Gastroenterol Hepatol 2022 20 2505 2.513E7 35835342 10.1016/j.cgh.2022.04.045

[8] Koh GE Ng B Lagström RMB Real-world assessment of the efficacy of computer-assisted diagnosis in colonoscopy: a single institution cohort study in Singapore Mayo Clin Proc Digit Health 2024 2 647 655 10.1016/j.mcpdig.2024.10.002 40206538 PMC11976013

[9] Lagström RMB Bräuner KB Bielik J Improvement in adenoma detection rate by artificial intelligence-assisted colonoscopy: Multicenter quasi-randomized controlled trial Endosc Int Open 2025 13 a25215169 10.1055/a-2521-5169 PMC11866038 40018072

[10] Kastenberg D Bertiger G Brogadir S Bowel preparation quality scales for colonoscopy World J Gastroenterol 2018 24 2833 2843 10.3748/wjg.v24.i26.2833 30018478 PMC6048432

[11] Hassan C Pickhardt PJ Rex DK A resect and discard strategy would improve cost-effectiveness of colorectal cancer screening Clin Gastroenterol Hepatol 2010 8 865 869 10.1016/j.cgh.2010.05.018 20621680

[12] Rex DK Ahnen DJ Baron JA Serrated lesions of the colorectum: review and recommendations from an expert panel Am J Gastroenterol 2012 107 1315 1329 22710576 10.1038/ajg.2012.161 PMC3629844

[13] Hyun E Helewa RM Singh H Serrated polyps and polyposis of the colon: a brief review for surgeon endoscopists Can J Surg 2021 64 E561 E566 10.1503/cjs.018820 34728521 PMC8565879

[14] Ishita B Paulina W Shin-ei K Real-time artificial intelligence–based optical diagnosis of neoplastic polyps during colonoscopy N Engl J Med 2022 1 EVIDoa2200003 10.1056/EVIDoa2200003 38319238

[15] Rex DK Bhavsar-Burke I Buckles D Artificial intelligence for real-time prediction of the histology of colorectal polyps by general endoscopists Ann Intern Med 2024 177 911 918 10.7326/M24-0086 38768450

[16] Hossain E Abdelrahim M Tanasescu A Performance of a novel computer-aided diagnosis system in the characterization of colorectal polyps, and its role in meeting Preservation and Incorporation of Valuable Endoscopic Innovations standards set by the American Society of Gastrointestinal Endoscopy DEN Open 2023 3 e178 10.1002/deo2.178 36320934 PMC9614381

[17] Baumer S Streicher K Alqahtani SA Accuracy of polyp characterization by artificial intelligence and endoscopists: a prospective, non-randomized study in a tertiary endoscopy center Endosc Int Open 2023 11 E818 E828 10.1055/a-2096-2960 37727511 PMC10506867

[18] Rondonotti E Hassan C Tamanini G Artificial intelligence-assisted optical diagnosis for the resect-and-discard strategy in clinical practice: the Artificial intelligence BLI Characterization (ABC) study Endoscopy Germany; 2023 55 14 22 10.1055/a-1852-0330 35562098

[19] Li JW Wu CCH Lee JWJ Real-world validation of a computer-aided diagnosis system for prediction of polyp histology in colonoscopy: a prospective multicenter study Am J Gastroenterol 2023 118 1353 1364 10.14309/ajg.0000000000002282 37040553

[20] Djinbachian R El Yamani MEM Rex DK Using computer-aided optical diagnosis and expert review to evaluate colorectal polyps diagnosed as normal mucosa in pathology Clin Gastroenterol Hepatol 2024 22 2344 23460 38705436 10.1016/j.cgh.2024.03.041

[21] Ponugoti P Rastogi A Kaltenbach T Disagreement between high confidence endoscopic adenoma prediction and histopathological diagnosis in colonic lesions ≤ 3 mm in size Endoscopy 2019 51 221 226 30722072 10.1055/a-0831-2348

[22] Shahidi N Rex DK Kaltenbach T Use of endoscopic impression, artificial intelligence, and pathologist interpretation to resolve discrepancies between endoscopy and pathology analyses of diminutive colorectal polyps Gastroenterology 2020 158 783 7850 31863741 10.1053/j.gastro.2019.10.024

[23] Rondonotti E Bergna IMB Paggi S White light computer-aided optical diagnosis of diminutive colorectal polyps in routine clinical practice Endosc Int Open 2024 12 E676 E683 10.1055/a-2303-0922 38774861 PMC11108657

[24] Weigt J Repici A Antonelli G Performance of a new integrated computer-assisted system (CADe/CADx) for detection and characterization of colorectal neoplasia Endoscopy 2022 54 180 184 10.1055/a-1372-0419 33494106

[25] Hassan C Rizkala T Mori Y Computer-aided diagnosis for the resect-and-discard strategy for colorectal polyps: a systematic review and meta-analysislancet Gastroenterol Hepatol 2024 9 1010 1019 10.1016/S2468-1253(24)00222-X 39303733

[26] Kim DH Fournier S Medawar E Prospective video-based study assessing effect of computer-assisted optical diagnosis on distinguishing serrated, hyperplastic, and adenomatous colorectal polyps Dig Dis Sci 2025 70 1477 1485 10.1007/s10620-025-08879-2 39946068

